# m^6^A reader protein YTHDF2 regulates spermatogenesis by timely clearance of phase‐specific transcripts

**DOI:** 10.1111/cpr.13164

**Published:** 2021-11-30

**Authors:** Meijie Qi, Haifeng Sun, Yueshuai Guo, Yu Zhou, Xueying Gu, Jiachuan Jin, Xiaoxu Chen, Fangzhu Wang, Honghui Ma, Xuejiang Guo, Hao Chen, Bin Shen

**Affiliations:** ^1^ State Key Laboratory of Reproductive Medicine Nanjing Medical University Nanjing China; ^2^ Center for Reproductive Medicine Division of Life Sciences and Medicine The First Affiliated Hospital of USTC University of Science and Technology of China Hefei China; ^3^ Reproductive Medicine Center Gansu Provincial Maternity and Child‐Care Hospital Lanzhou China; ^4^ Department of Cardiology Shanghai East Hospital Tongji University School of Medicine Shanghai China; ^5^ Department of Human Cell Biology and Genetics School of Medicine Southern University of Science and Technology Shenzhen China; ^6^ Gusu School Nanjing Medical University Nanjing China; ^7^ Center for Global Health School of Public Health Nanjing Medical University Nanjing China; ^8^ Women’s Hospital of Nanjing Medical University Nanjing Maternity and Child Health Care Hospital Nanjing Medical University Nanjing China

**Keywords:** m^6^A, male sterility, mRNA clearance, spermatogenesis, *Ythdf2*

## Abstract

**Objectives:**

Accumulating evidences show that the regulatory network of m^6^A modification is essential for mammalian spermatogenesis. However, as an m^6^A reader, the roles of YTHDF2 remain enigmatic due to the lack of a proper model. Here, we employed the germ cell conditional knockout mouse model and explored the function of YTHDF2 in spermatogenesis.

**Materials and methods:**

*Ythdf2* germ cell conditional knockout mice were obtained by crossing *Ythdf2*‐floxed mice with *Vasa*‐*Cre* and *Stra8*‐*Cre* mice. Haematoxylin and eosin (HE) staining, immunofluorescent staining and Western blotting were used for phenotyping. CASA, IVF and ICSI were applied for sperm function analysis. RNA‐seq, YTHDF2‐RIP‐seq and quantitative real‐time PCR were used to explore transcriptome changes and molecular mechanism analysis.

**Results:**

Our results showed that YTHDF2 was highly expressed in spermatogenic cells. The germ cell conditional knockout males were sterile, and their sperm displayed malformation, impaired motility, and lost fertilization ability. During differentiated spermatogonia transiting to pachytene spermatocyte, most m^6^A‐modified YTHDF2 targets that were degraded in control germ cells persisted in pachytene spermatocytes of *Ythdf2*‐vKO mice. These delayed mRNAs were mainly enriched in pathways related to the regulation of transcription, and disturbed the transcriptome of round spermatid and elongated spermatid subsequently.

**Conclusion:**

Our data demonstrate that YTHDF2 facilitates the timely turnover of phase‐specific transcripts to ensure the proper progression of spermatogenesis, which highlights a critical role of YTHDF2 in spermatogenesis.

## Graphical abstract

YTHDF2 accelerates the degradation of m^6^A‐modified RNA during the early stages of spermatogenesis. The timely clearance of phase‐specific transcripts mediated by YTHDF2 ensures the formation of functional sperm.

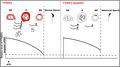

## 1 INTRODUCTION

N6‐methyladenosine (m^6^A), the most abundant chemical modification in mRNA, plays crucial roles in multiple biological processes.^1^, ^2^ The mRNA m^6^A modification is dynamic and reversible. m^6^A is mainly installed by METTL3 and METTL14 core complex, together with several other key components, including WTAP, VIRMA and ZC3H13,^3^, ^4^, ^5^, ^6^ and this modification can also be removed by the ‘eraser’ proteins ALKBH5 and FTO.^7^, ^8^ Importantly, m^6^A exerts its regulatory functions primarily through various ‘readers’, including YTH domain‐containing proteins (YTHDF1, YTHDF2, YTHDF3, YTHDC1 and YTHDC2), eIF3, HnRNP and IGF2BP2.^9^, ^10^, ^11^, ^12^ In different biological contexts, the reader proteins specifically decipher the m^6^A‐decorated RNAs by recruiting distinct effectors, which are directly involved in regulating RNA stability, translation, splicing and subsequently determining the fates of m^6^A‐containing transcripts.^9^, ^13^, ^14^, ^15^, ^16^

Accumulating evidences showed the regulatory network of m^6^A modification is essential for mammalian spermatogenesis. For instance, germ cell–specific inactivation of METTL3 or METTL14 induces progressive loss of spermatogonial stem cells (SSC).^17^, ^18^ Depletion of ALKBH5 results in abnormal spermatogenesis, due to aberrant metabolism of m^6^A‐marked mRNAs.^7^, ^19^ Previous reports demonstrated that different ‘reader’ proteins are also critical for spermatogenesis by recognizing m^6^A marks on specific mRNAs and mediating various processes of RNA metabolism. Among the m^6^A reader proteins, YTHDC1 was uncovered to be essential for SSC survival,^20^ and YTHDC2, proved to be an m^6^A ‘reader’ by our previous research,^21^ targets meiosis‐related genes and its loss induces defects in meiosis.^21^, ^22^, ^23^, ^24^ Besides YTHDC1 and YTHDC2, the abundant expression of YTHDF1, YTHDF2 and YTHDF3 can also be detected in testis, implying that these genes may be functional during spermatogenesis. However, ablation of *Ythdf1* or *Ythdf3* does not cause noticeable defects in spermatogenesis.^25^ As to *Ythdf2*, its exact roles are controversial in different studies. An initial study claimed that knockout of *Ythdf2* does not affect the fertility of male mice,^26^ but a more recent study showed that deletion of *Ythdf2* leads to male hypofertility.^25^

To clarify the biological roles and underlying mechanism of YTHDF2 during spermatogenesis, in this study, we generated a conditional knockout mouse model with a deletion of the critical domain of YTHDF2, and revealed that YTHDF2 is indispensable for spermatogenesis and deletion of *Ythdf2* in male germ cells resulted in sterility phenotype. Mechanistically, YTHDF2 facilitated the timely turnover of phase‐specific transcripts to ensure the proper progression of spermatogenesis.

## 2 MATERIALS AND METHODS

### 2.1 *Ythdf2* cKO mouse generation

*Ythdf2*‐floxed mice were generated before by our laboratory.^27^, ^28^ We generated germ cell–specific knockout mice by crossing *Ythdf2*‐floxed mice with *Vasa*‐Cre or *Stra8*‐Cre mice,^18^, ^29^ respectively. All mice used in this study were kept at C57BL/6 genetic background, and housed under specific pathogen‐free (SPF) conditions.

### 2.2 Histological and immunofluorescent staining analysis

For haematoxylin and eosin (H&E) staining, testes were fixed in modified Davidson's fluid (MDF), dehydrated with increasing concentration of ethanol, embedded in paraffin and cut into 5‐μm‐thick sections. Then, the sections were deparaffinized, rehydrated and stained with haematoxylin and eosin.

For immunofluorescent staining, the sections were deparaffinized, rehydrated and boiled in sodium citrate buffer (Maxim, MVS‐0066) for 10 min. After washing three times by 0.1% Triton ×‐100 in PBS, the sections were blocked with 5% BSA and then incubated with primary antibody overnight at 4°C. Primary antibodies used in this study were as follows: anit‐YTHDF2 (Abcam, ab220163), anti‐SYCP3 (Abcam, ab15093), and anti‐γH2AX (Abcam, ab26350). On the next day, the slides were washed three times in PBST (0.1% Tween‐20 in PBS) and incubated with the secondary antibody and Hoechst at room temperature for 2 h. Finally, the slides were washed in PBST and mounted with 50% glycerol.

For spermatocyte chromosome spreads, seminiferous tubules were placed in hypotonic extraction buffer (30 mM Tris‐HCl pH 8.5, 50 mM sucrose, 17 mM citric acid, 5 mM EDTA, 2.5 mM DTT and 1 mM PMSF) for 45 min at room temperature. Then, the tissues were minced in 100 mM sucrose. 10 μl of cell suspension was pipetted onto slides and fixed by fixative (1% PFA, 0.15% Triton ×‐100, 10 mM sodium borate) for 3 h. After washing four times by TBS (150 mM NaCl, 20 mM Tris‐HCl, pH 7.6), samples were blocked with blocking buffer (1% normal donkey serum, 0.3% BSA and 0.05% Triton ×‐100) for 1 h at room temperature, and incubated with primary antibodies overnight at 37°C. On the next day, after washing 3 times by TBS, slides were blocked with blocking buffer for 5 h, and finally incubated with secondary antibodies for 1.5 h at 37°C. After washing three times by TBS, the slides were mounted with 50% glycerol.

### 2.3 TUNEL assay

TUNEL assay was performed with TUNEL BrightRed Apoptosis Detection Kit (Vazyme, A113‐01) according to the manufacturer's instructions. Briefly, the paraffin sections were deparaffinized, rehydrated and incubated with PBS containing Proteinase K at room temperature for 20 min. After washing two times by PBS, the sections were incubated with equilibration buffer at room temperature for 30 min, and TdT buffer at 37°C for 1 h. Then, the sections were washed three times by PBS and incubated with DAPI at room temperature for 5 min. The slides finally were washed by PBS and mounted with 50% glycerol.

### 2.4 Computer‐assisted sperm analysis

Sperm was collected from cauda epididymides and incubated with HTF medium (Irvine Scientific, 90125) for 5 min at 37°C. 10 μl of sperm suspension was placed in slide chamber and analysed by IVOS II system (Hamilton Thorne) with default parameters.

### 2.5 In vitro fertilization (IVF) and intracytoplasmic sperm injection (ICSI)

For IVF, sperm was harvested from cauda epididymides of control and vKO mice aged 10–20 weeks and incubated in HTF medium for 1 h. Cumulus–oocyte complexes (COCs) were collected from oviduct ampullae of wild‐type mice and incubated in HTF drops. Then, the sperm was added to HTF drops containing COCs and incubated at 37°C for 5 h. Finally, the presumptive zygotes were washed several times until the cumulus cells and excess sperm were removed. Then, the zygotes were transferred into KSOM medium (Millipore, MR‐020P‐D) and cultured in a humidified atmosphere at 37°C with 5% CO_2_.

For ICSI, sperm was isolated from cauda epididymides of control and vKO mice aged 10–20 weeks and incubated in HTF medium for 1 h. COCs were isolated from oviduct ampullae of wild‐type mice, followed by removing the cumulus cells in medium containing 0.5 mg/ml hyaluronidase (Sigma, H3506) at 37°C. Then, the dispersed sperm was injected into the MII oocyte by a microinjector. The 1‐cell development rate is calculated by dividing the number of zygotes by the number of injected MII oocytes. The 2‐cell or blastocyst rate is defined as the ratio of 2‐cell or blastocyst to the number of zygotes.

### 2.6 Western blot analysis

Total protein lysates were extracted with RIPA lysis buffer. Then, the protein samples were loaded and separated in a SDS‐PAGE gel, transferred onto the PVDF membranes (Millipore, IPVH00010). The membranes were blocked with 5% skimmed milk for 2 h, and then incubated with primary antibodies overnight at 4°C. On the next day, the membranes were washed four times using TBST and incubated with secondary antibody at room temperature for 2 h. Primary antibodies were as follows: anti‐GAPDH (Santa Cruz, sc‐32233) and anti‐YTHDF2 (Abcam, ab220163).

### 2.7 Isolation of spermatogenic cells

The STA‐PUT method was used to isolate pachytene spermatocyte, round spermatid and elongated spermatid as described previously.^30^ Briefly, mouse testes were decapsulated and digested with collagenase IV (1 mg/ml) until the seminiferous tubules were dispersed. The dispersed seminiferous tubules were collected by centrifugation at 500 g. The pellet was further digested with 0.25% trypsin and filtered through a 40‐μm Nylon Cell Strainer. The cell suspension was bottom‐loaded into a cell separation apparatus, followed by a 2%–4% bovine serum albumin (BSA) gradient. Then, the cell fractions were harvested after about 2.5 h of sedimentation. Magnetic‐activated cell sorting (MACS) was used to isolate the undifferentiated and differentiating spermatogonia. Undifferentiated spermatogonia were purified using CD90.2 Positive Selection Kit II (STEM CELL, #19851) from P6.5 testes. Differentiating spermatogonia were purified using CD117 (cKIT) Positive Selection Kit (STEM CELL, #18757) from P10.5 testes.

### 2.8 RNA extraction and qRT‐PCR

Total RNA was extracted using RNAiso Plus (TaKaRa, #9109) according to the manufacturer's instructions. cDNA synthesis was carried out using the PrimeScript RT reagent Kit with gDNA Eraser (TaKaRa, RR047B). Real‐time RT–PCR analysis was performed using AceQ qPCR SYBR Green Master Mix (Vazyme, Q141‐03). Primer sequences are listed in Table S1.

### 2.9 RNA‐seq and data analysis

For bulk RNA‐seq libraries, total RNA was extracted with TRIzol from purified germ cells. mRNA libraries were prepared with VAHTS mRNA‐seq V3 Library Prep Kit for Illumina (Vazyme, NR611) according to the manufacturer's instructions, and sequenced on Illumina NovaSeq platform.

Quality control, removal of overrepresented sequences and sequencing adapters were performed with FastQC and Trim Galore, respectively. Filtered reads were aligned to GRCm38 using HISAT2 (version 2.2.1) with default parameters. Then, the gene expression level (read counts) was quantified by featureCounts (version 1.6.0). Differential expression testing was performed with R package DESeq2 (version 1.38.0). Genes with false discovery rate (FDR)–adjusted *p*‐value <0.05 and fold change >1.5 were marked as differentially expressed genes (DEGs). Gene ontology (GO) enrichment analysis was performed using DAVID. DEGs are listed in Table S2.

### 2.10 YTHDF2‐RIP‐seq and data analysis

Whole testes or purified germ cells were lysed with lysis buffer (150 mM KCl, 20 mM Tris‐HCl, pH 7.5, 2 mM EDTA, 0.5% NP‐40, 0.5 mM DTT, 1:100 protease inhibitor cocktail and 1:100 SUPERase•In RNase Inhibitor) for 30 min with gentle rotary shaking at 4°C, and then centrifuged at 16,000 *g* for 20 min. The supernatant was pre‐cleared with Dynabeads protein A/G beads for 1 h at 4°C. Meanwhile, 100 μl of protein A/G beads was coated with YTHDF2 antibody (ProteinTech, 24744–1‐AP) for 1 h at 4°C. 1/10 of pre‐cleared samples were saved as input, and the rest samples were incubated with the pre‐coated beads for 4 h at 4 °C. After washing 6 times with NT2 buffer (200 mM NaCl, 2 mM EDTA, 0.05% NP‐40, 50 mM Tris‐HCl pH 7.5, 0.5 mM DTT, 1:1 000 protease inhibitor and 1:1 000 SUPERase•In RNase Inhibitor), the RNAs bound to the Dynabeads were extracted with TRIzol. The recovered and input RNAs were used to generate RNA‐seq libraries following the Smart‐seq2 protocol as described previously.^31^

Quality control of sequencing data was performed by FastQC and Trim Galore. FPKM was calculated by using RSEM with default parameters. The RIP target was defined as Input FPKM >1 and IP FPKM / Input FPKM >1.2. The intersection of RIP targets between two replicates was identified as the final target genes of YTHDF2. YTHDF2‐targeted genes are listed in Table S3.

The intersection of the following four data sets was identified as delayed‐decay RNAs upon YTHDF2 depletion: (1) the significant down‐regulated genes (adjusted *p* values <0.05 and fold change >1.5) between two development stages of wild‐type germ cells (such as SG to P period or P to RS period); (2) the m6A‐modified genes in wild‐type germ cells of previous stage (these data were acquired from the published data^18^); (3) the YTHDF2‐targeted genes; and (4) the genes with an up‐regulated tendency between two stages in *Ythdf2*‐null mice (fold change >1.2).

## 3 RESULTS

### 3.1 YTHDF2 in germ cells is essential for male fertility

**FIGURE 1:**
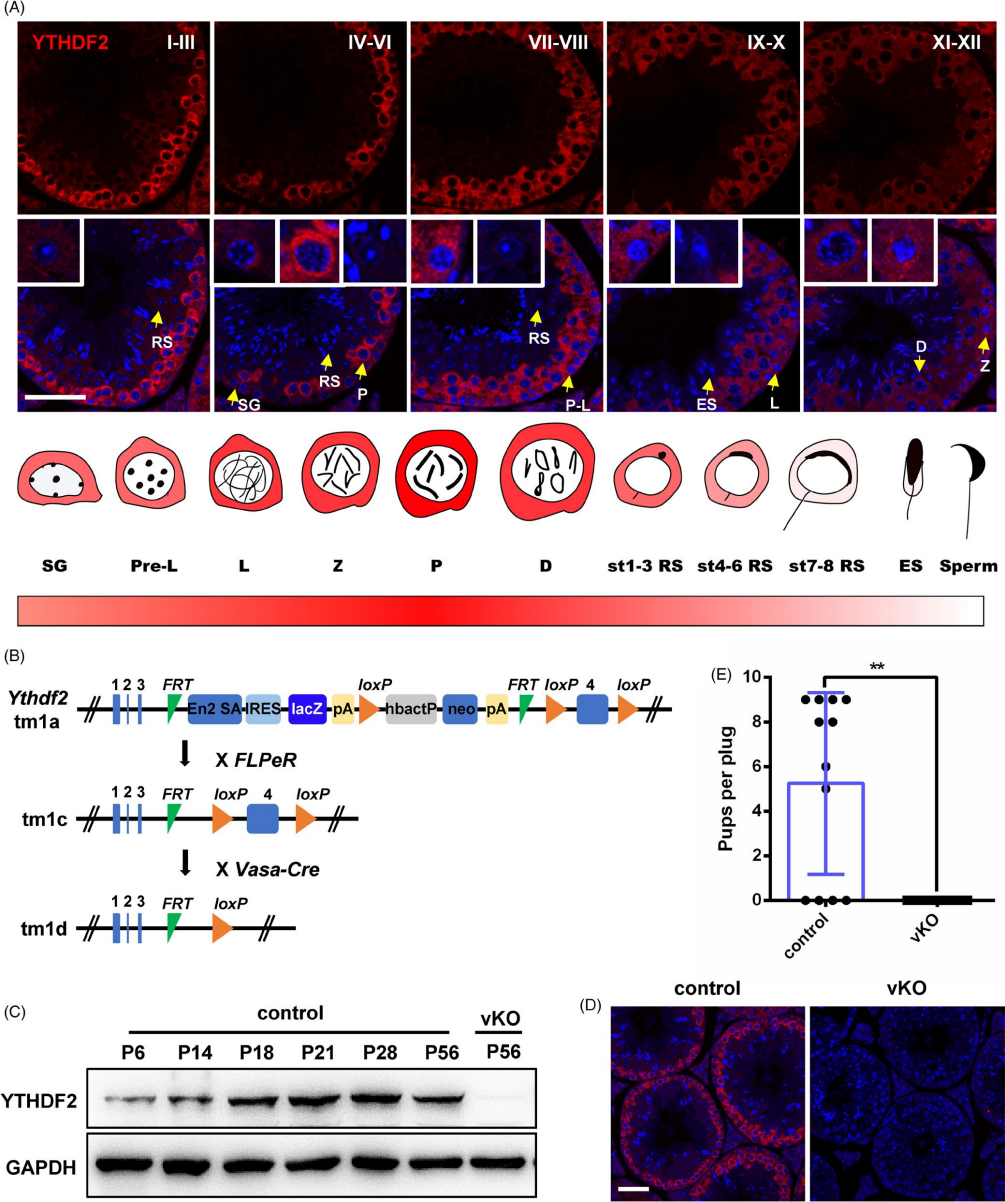
YTHDF2 is essential for male mouse fertility. (A) Immunofluorescent staining of YTHDF2 in adult testis. Scale bar, 50 μm. I‐XII indicates the cycle of the seminiferous epithelium. SG, spermatogonia; Pre‐L, pre‐leptotene spermatocyte; L, leptotene spermatocyte; Z, zygotene spermatocyte; P, pachytene spermatocyte; D, diplotene spermatocyte; RS, round spermatid; ES, elongated spermatid; st, stage. (B) Schematic diagram for the *Ythdf2* targeting strategy. (C) Western blot analysis of YTHDF2 in control and *Ythdf2*‐vKO testes at different ages. P, postnatal day. (D) Immunofluorescent staining of YTHDF2 in control and *Ythdf2*‐vKO testes. Scale bar, 50 μm. (E) Litter size per plug from *Ythdf2*‐vKO and control mice. Data are presented as means ± SD (*n* ≥ 9 for each group). Significance was calculated with unpaired two‐tailed Student's *t*‐test (** *p *< 0.01)

Considering that *Ythdf2* is highly and dynamically expressed in male germ cells^29^ (Figure S1A), we postulated that YTHDF2 likely plays functional roles during spermatogenesis. Immunofluorescent staining showed that YTHDF2 was mainly located in the cytoplasm of germ cells and its protein level was highest in spermatocytes, followed by spermatogonia, and lowest in haploid germ cells (Figure 1A). To determine the function of YTHDF2 in spermatogenesis, we generated *Ythdf2*‐KO first mouse by flanking the exon 4 with *loxP* sites, which encodes the critical YTH domain of YTHDF2 (Figure 1B and Figure S1B). By mating with FLPeR mice, sequentially with *Vasa*‐*Cre* transgenic mouse (Cre recombinase is initially expressed in primordial germ cells), *Ythdf2* germ cell–specific knockout mice were obtained (hereafter referred to as *Ythdf2*‐vKO) (Figure 1B). Western blot and immunofluorescence analyses confirmed the germ cell–specific depletion of YTHDF2 in *Ythdf2*‐vKO testes (Figure 1C,D). In addition, the onset of high expression of YTHDF2 in P18 testis also indicated that it was mostly enriched in spermatocytes (Figure 1C). We then sought to investigate the fertility of *Ythdf2*‐vKO male mice by mating them with wild‐type females. Although copulation plugs could be observed normally, no offspring of *Ythdf2*‐vKO male mice was born (Figure 1E), suggesting that knockout of *Ythdf2* in germ cells caused loss of male fertility.

### 3.2 *Ythdf2* deficiency leads to sperm defects and loss of fertilization capacity

**FIGURE 2:**
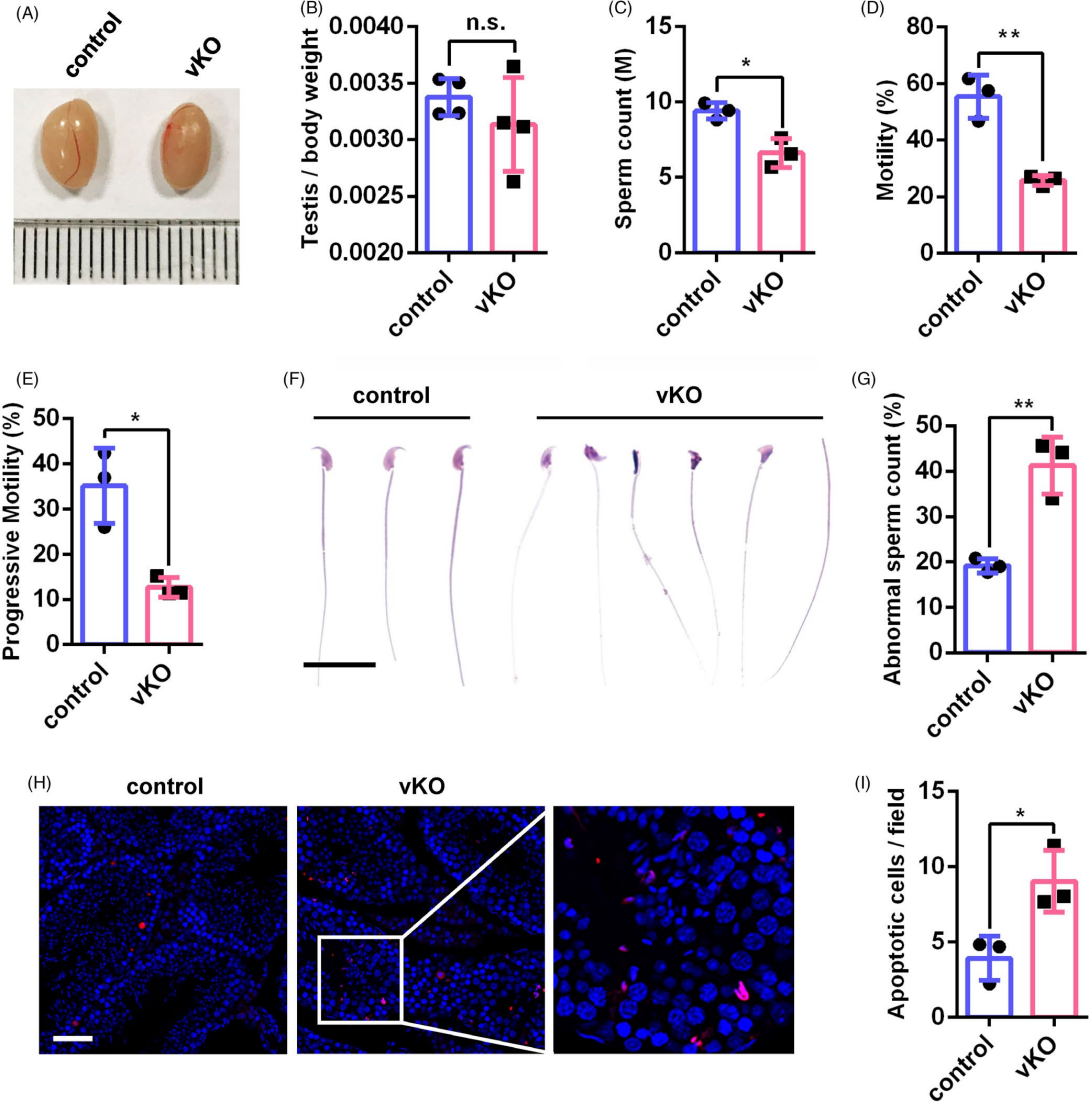
*Ythdf2* deletion results in sperm defects. (A) Gross morphology of representative testes from adult control and age‐matched *Ythdf2*‐vKO. (B) The testis/body weight ratio in adult control and *Ythdf2*‐vKO mice. (C) The sperm count in cauda epididymides of adult control and age‐matched *Ythdf2*‐vKO mice. M, million. (D) and (E) CASA assay of motility and progressive motility of sperm from adult control and age‐matched *Ythdf2*‐vKO mice. (F) Haematoxylin and eosin‐stained sperm collected from control and *Ythdf2*‐vKO cauda epididymides. Scale bar, 20 μm. (G) The percentage of abnormal sperm in adult control and *Ythdf2*‐vKO mice. (H) TUNEL assay of adult control and age‐matched *Ythdf2*‐vKO testes. Scale bar, 50 μm. (I) Quantification of apoptotic cells in adult control and age‐matched *Ythdf2*‐vKO testes. Data are presented as means ± SD (B, *n *= 4 for each group; C‐E, G and I, *n* = 3 for each group). Significance was calculated with unpaired two‐tailed Student's *t*‐test (n.s., not significant, * *p* < 0.05, ** *p *< 0.01)

To examine the male infertility of *Ythdf2*‐vKO mice in detail, we first analysed the gross morphology of testes. No noticeable difference was observed in testis size and weight between *Ythdf2*‐vKO and control mice (Figure 2A,B). Then, we performed computer‐assisted sperm analysis (CASA) and found that the count of epididymal sperm significantly reduced in *Ythdf2*‐vKO mice (Figure 2C). CASA result showed that the motility and progressive motility of *Ythdf2*‐vKO sperm were markedly impaired (Figure 2D,E). Histological analysis of sperm collected from cauda epididymides indicated that more than 40% of sperms were deformed with various structural abnormalities (Figure 2F,G and Figure S2A). TUNEL assay revealed that the apoptotic cells increased significantly in *Ythdf2*‐vKO testes compared with the control (Figure 2H,I), and the apoptosis signal was mainly detected in elongated spermatids, suggesting that these sperm cells may be retained and phagocytosed by Sertoli cells (Figure 2H).

To investigate whether the *Ythdf2*‐vKO sperm is functionally normal in fertilization, in vitro fertilization (IVF) was carried out and these sperm failed to fertilize the wild‐type oocytes (Figure S2B). Interestingly, intracytoplasmic sperm injection (ICSI) of *Ythdf2*‐vKO sperm showed that fertilized eggs were able to develop to blastocytes normally, in spite of lower fertilization rates of *Ythdf2*‐null sperm than that of wild‐type sperm (Figure S2C, D). These results suggested that *Ythdf2* deficiency impaired the natural fertilization ability of sperm.

Since YTHDF2 is highly expressed in spermatogonia and spermatocytes, we reasoned that *Ythdf2*‐vKO mice might display abnormalities in the early stage of spermatogenesis. Unexpectedly, *Ythdf2*‐vKO mice showed normal spermatogenesis cycle (Figure S3A). Then, we performed chromosome spreads of spermatocytes and immunofluorescent staining with γH2AX (DNA double strand break marker) and SYCP3 (an axial element protein of synaptonemal complex) to examine the meiosis process. The results indicated that the expression and localization of γH2AX and SYCP3 appeared normal at the four stages (leptotene, zygotene, pachytene and diplotene) of *Ythdf2*‐vKO spermatocytes (Figure S3B), and there was no significant difference in the ratio of the four kinds of spermatocytes compared with the control mice (Figure S3C), suggesting that the absence of YTHDF2 has no obvious effect towards meiosis.

A previous study reported that YTHDF2 regulates the migration and proliferation of GC‐1 spermatogonial cell line.^32^ Then, we asked whether YTHDF2 functions in spermatogonia in vivo. Immunofluorescent staining indicated that there is no noticeable difference in the number of PLZF‐positive spermatogonia between *Ythdf2*‐vKO and control mice (Figure S4A, B), implying that YTHDF2 is unlikely to be essential for spermatogonia. To further narrow down the exact stage when YTHDF2 regulates spermatogenesis, we crossed *Ythdf2*‐floxed mice with *Stra8*‐Cre mice, which induces recombination starting from type A1 spermatogonia, and obtained a *Ythdf2*‐null model before meiosis initiation (*Ythdf2*‐sKO). As expected, immunofluorescent staining result demonstrated that YTHDF2 was still readily detected in spermatogonia, while it disappeared thereafter in *Ythdf2*‐sKO testes (Figure S5A). Similar to *Ythdf2*‐vKO mice, although the overall morphology of *Ythdf2*‐sKO testis was comparable with the control (Figure S5B, C), the sperm count, motility and progressive motility in *Ythdf2*‐sKO were all substantially decreased (Figure S5D‐F), along with the increased number of deformed sperm and loss of fertilizing capacity (Figure S5G‐I). The TUNEL result also revealed that the apoptotic sperm in the testes of *Ythdf2*‐sKO mice increased significantly (Figure S5J).

Taken together, these results demonstrate that *Ythdf2* deficiency apparently does not affect the early stage of spermatogenesis, but leads to sperm defects and loss of fertilization capacity.

### 3.3 YTHDF2 depletion in germ cells disturbs transcriptome during spermatogenesis

**FIGURE 3:**
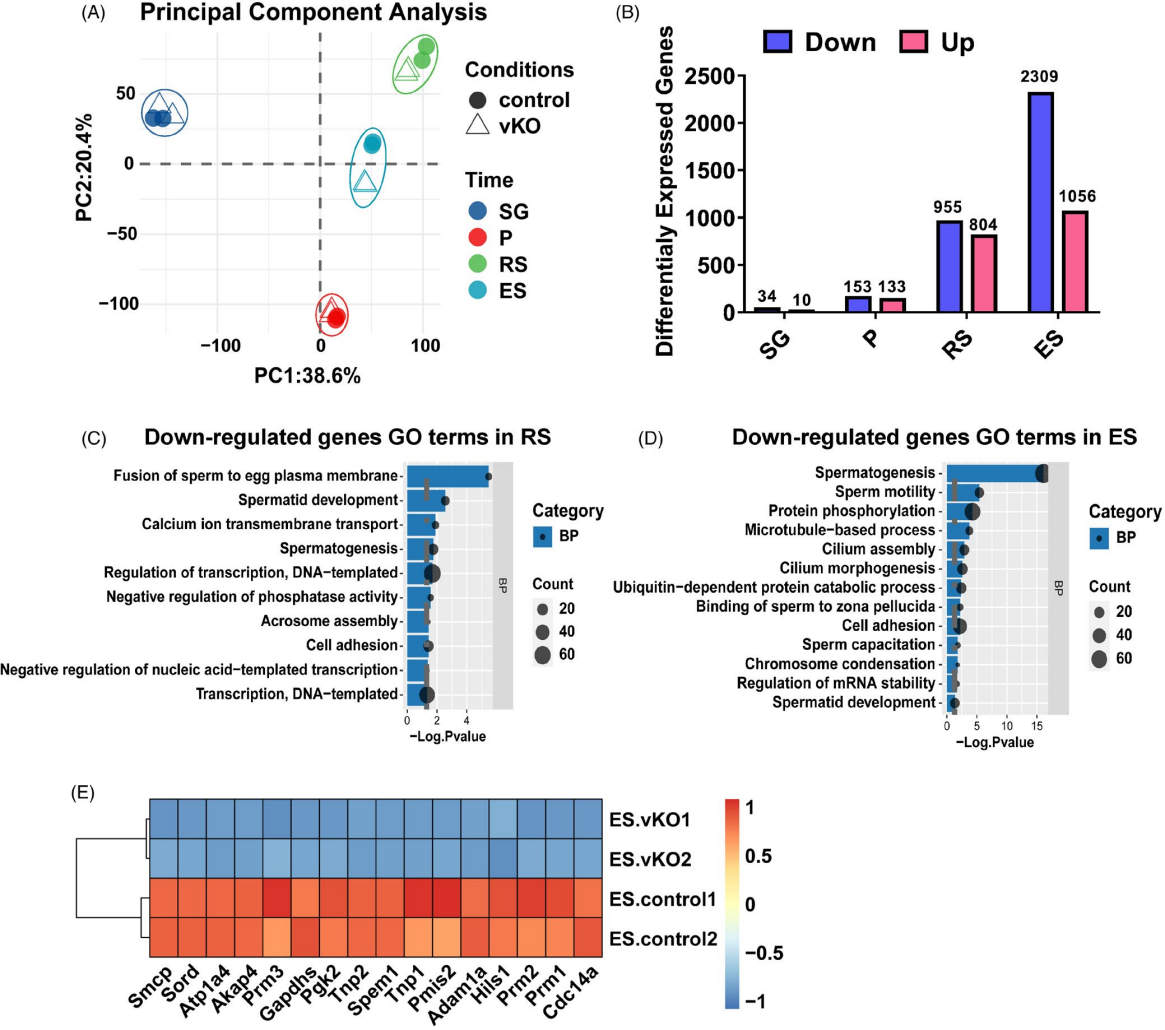
YTHDF2 depletion disrupts transcriptome in late spermatogenesis. (A) PCA of transcriptome from various stages of purified germ cells. (B) The number of dysregulated genes in various stages of purified germ cells. (C) GO analysis of the down‐regulated genes in round spermatids of *Ythdf2*‐vKO mice. (D) GO analysis of the down‐regulated genes in elongated spermatids of *Ythdf2*‐vKO mice. (E) Expression of DEGs in elongated spermatids of control and *Ythdf2*‐vKO mice

To explore the underlying mechanism of sperm defects upon YTHDF2 depletion, we systematically illuminated genome‐wide gene expression changes induced by *Ythdf2* deletion in germ cells. Firstly, we isolated undifferentiated spermatogonia, differentiated spermatogonia, pachytene spermatocytes, round spermatids and elongated spermatids. Then, we performed RNA sequencing of the purified germ cells and gene expression analysis. The result demonstrated that the transcriptomic profiles of various stages of germ cells from control mice were comparable to the previous report,^33^ indicating a high purity of the isolated germ cells (Figure S6A). Principal component analysis (PCA) of gene expression showed the transcriptome diverged along with spermatogenesis between control and *Ythdf2*‐vKO mice (Figure 3A). Only a few number of genes are altered in undifferentiated and differentiated spermatogonia of *Ythdf2*‐vKO compared with the control mice (Figure 3B and Figure S6B, C), consistent with our above observation that *Ythdf2* deficiency does not influence the self‐renewal and differentiation of spermatogonia (Figure S4A, B). After pachytene stage, the number of differentially expressed genes (DEGs) between *Ythdf2*‐vKO and control germ cells steadily increased (Figure 3B and Figure S6D‐F). For instance, in differentiated spermatogonia, loss of YTHDF2 does not cause considerable transcriptomic changes, while in the elongated spermatid, there were as many as 1056 genes up‐regulated and 2309 genes down‐regulated (Figure 3B). Gene ontology (GO) analysis showed that the down‐regulated DEGs in round spermatid were significantly enriched in sperm‐egg fusion, spermatid development and acrosome assembly (Figure 3C); in elongated spermatid, the down‐regulated DEGs were mainly associated with sperm motility, cilium assembly, binding of sperm to zona pellucida and chromosome condensation (Figure 3D). Many DEGs in elongated spermatid have been reported to be closely related to spermiogenesis. For example, *Smcp*, *Sord*, *Atp1a4*, *Cdc14a* and *Pgk2* are all important for sperm motility^34^, ^35^, ^36^, ^37^, ^38^; *Akap4* and *Spem1* deletion results in sperm deformity^39^, ^40^; and *Pmis2* null sperm fails to bind to the zona pellucida^41^ (Figure 3E). Together, the transcriptome of *Ythdf2*‐vKO germ cells changed mainly thereafter pachytene stage, and the DEGs were coincident with the sperm malformation, impaired motility and loss of fertilization capacity in *Ythdf2*‐vKO mice.

### 3.4 YTHDF2 is required for m^6^A‐modified mRNA clearance

**FIGURE 4:**
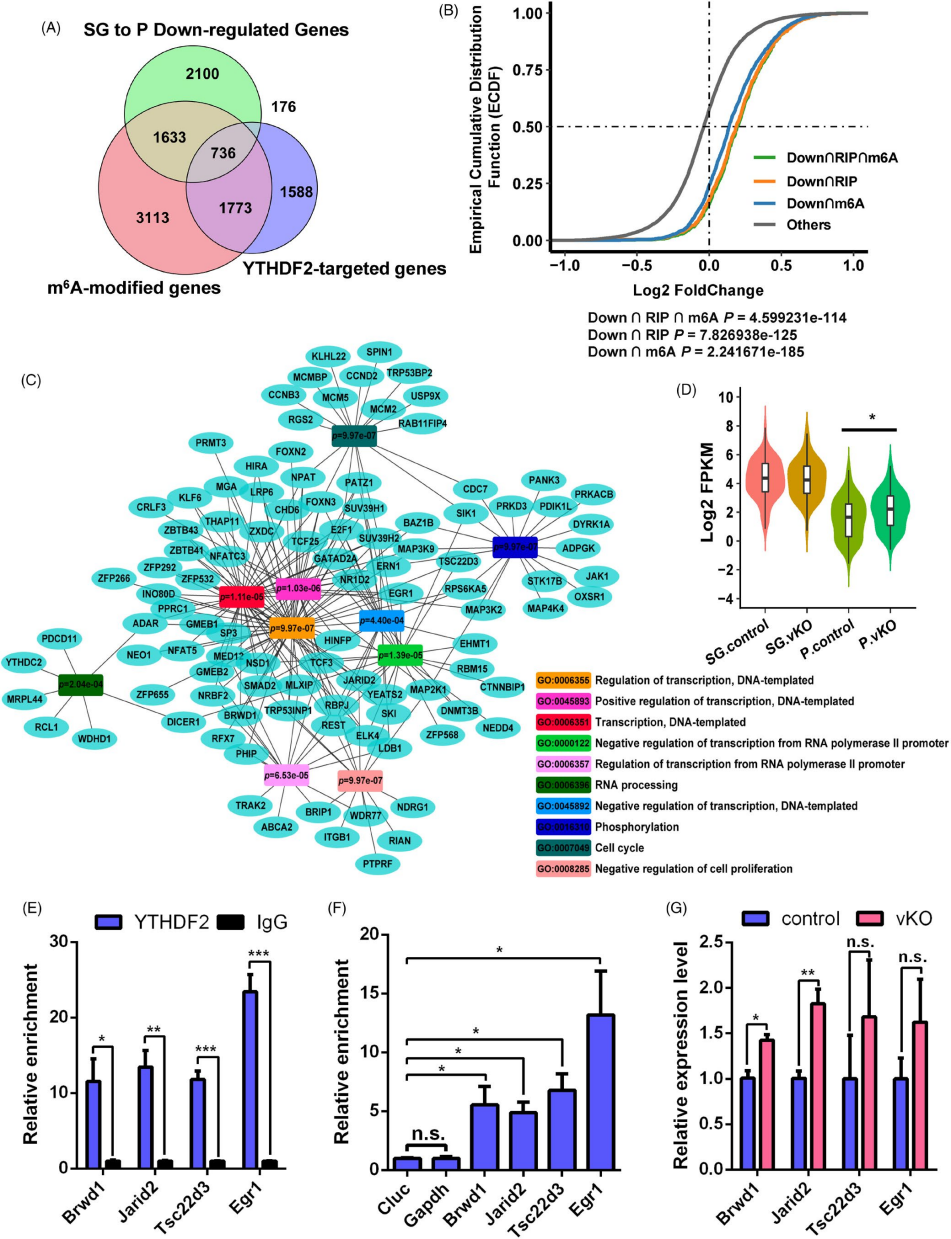
YTHDF2 promotes the degradation of its target mRNA. (A) Venn diagram showing that overlaps among genes down‐regulated from control differentiated spermatogonia to pachytene spermatocyte, m^6^A‐modified genes and YTHDF2‐targeted genes. The m^6^A‐seq data were acquired from a published data^18^. YTHDF2‐targeted genes were identified in adult whole testes. (B) Cumulative distributions of relative stability change between control and *Ythdf2*‐vKO pachytene spermatocytes. (C) GO analysis of genes with delayed degradation from differentiated spermatogonia to pachytene spermatocyte. (D) The RNA expression level of transcription‐associated genes at different stages of germ cells. Significance was calculated with two‐tailed Mann‐Whitney test (*p < 0.05). (E) YTHDF2‐targeted candidates verified by YTHDF2‐RIP‐qPCR in control and *Ythdf2*‐vKO testes. IgG was used as negative control. (F) m^6^A‐enriched candidates verified by m^6^A‐RIP‐qPCR in control and *Ythdf2*‐vKO testes. Non‐m^6^A‐modified gene Cluc was used as a negative control. (G) qPCR validation of delayed genes in control and *Ythdf2*‐vKO pachytene spermatocytes. Data of (E‐G) are presented as means ± SEM (*n* = 3 for each group). Significance was calculated with unpaired two‐tailed Student's *t*‐test (n.s., not significant, *p < 0.05, **p < 0.01, ***p < 0.001)

Previous studies have shown that YTHDF2 preferentially recognizes m^6^A‐modified mRNA and mediates their rapid degradation in many biological contexts.^27^, ^42^, ^43^, ^44^, ^45^ Based on the expression pattern of YTHDF2 and the phenotype of *Ythdf2*‐vKO mice, we focused on gene expression changes in stages from differentiated spermatogonia‐to‐pachytene spermatocyte transition, and pachytene spermatocyte‐to‐round spermatid transition. Transcriptomic analysis showed that there were 4645 genes down‐regulated during the transition from differentiated spermatogonia to pachytene spermatocyte (Figure 4A), and there were 7041 genes down‐regulated during the transition from pachytene spermatocyte to round spermatid (Figure S8B). To explore the biological mechanism of YTHDF2 in testis, we carried out YTHDF2‐RIP‐seq to identify the direct targets of YTHDF2. The correlation of two replicates of YTHDF2‐targeted transcripts from adult whole testes and pachytene spermatocytes was high positive and moderate positive, respectively (Figure S7A, E). The YTHDF2 enrichment genes were comparable between two replicates (Figure S7B‐D, F‐H), indicating that the targets of YTHDF2 we obtained were reliable. The RIP‐seq of whole testes identified 4273 YTHDF2‐targeted transcripts, among which 2509 targets were m^6^A‐modified (Figure 4A). RIP‐seq of pachytene spermatocytes identified 3012 YTHDF2‐targeted transcripts, of which 2063 targets were also marked with m^6^A (Figure S8B).

We next sought to compare the degradation rates of YTHDF2‐targeted mRNA during spermatogenesis. During differentiated spermatogonia transiting to pachytene spermatocyte, 736 m^6^A‐modified YTHDF2 targets were degraded in control germ cells (Figure 4A), while in *Ythdf2*‐vKO germ cells, almost all of them persisted in pachytene spermatocytes, with 233 genes fold change >1.2 (Figure 4B and Figure S8A). Remarkably, GO analysis showed that these delayed mRNAs were mainly enriched in pathways related to the regulation of transcription (Figure 4C), and the transcription‐associated genes showed a higher level in *Ythdf2*‐vKO than that in control mice (Figure 4D). Among the aberrant degradation of transcription‐associated genes, such as *Brwd1*, *Jarid2*, *Egr1* and *Tsc22d3*, all of these genes were m6A‐modified and YTHDF2‐targeted, and they could be independently validated by real‐time qPCR (Figure 4E‐G). Interestingly, BRWD1 contains a Bromodomain and is closely related to chromatin remodelling^46^; JARID2 is a transcription repressor that recruits Polycomb repressive complex 2 (PRC2) to regulate gene expression.^47^ The delayed degradation of these transcription‐associated genes may cause abnormal transcription in the subsequent stages via a transcriptional cascade effect.^48^ Indeed, from pachytene spermatocyte to elongated spermatid, we observed a 10.8‐fold increase (286 to 3365) in the number of DEGs (Figure 3B).

In the transition from pachytene spermatocytes to round spermatid, 1481 m^6^A‐modified YTHDF2 targets were degraded (Figure S8B), and 389 of them showed delayed decay in *Ythdf2*‐vKO round spermatid (Figure S8C, D). GO analysis showed that most of these delayed mRNAs were engaged in cell cycle and transcription (Figure S8E). Although YTHDF2 is more likely involved in this process based on its expression pattern and the phenotype of *Ythdf2*‐vKO mice, we cannot rule out the possibility that these alterations were caused indirectly, because a large number of transcription‐related factors have been abnormally expressed in the previous stage. Considering that YTHDF2 is still low expressed in round spermatid, we intended to explore whether the DEGs in round spermatid are directly regulated by YTHDF2 via m^6^A modification. Only 8.27% (79/955) down‐regulated genes and 4.48% (36/804) up‐regulated genes were both YTHDF2‐targeted and m6A‐modified (Figure S9A, B), and these genes had no obvious relationship with round spermatid development, suggesting YTHDF2 may have no direct effect on round spermatid development.

In summary, the improper accumulation of mRNA induced by YTHDF2 depletion may be the cause of abnormalities in the later spermatogenesis stages, ultimately leading to male sterility.

## 4 DISCUSSION

m^6^A is the most prevalent modification of eukaryotic mRNA.^49^ It has been reported to be involved in almost every aspect of RNA metabolism, including mRNA splicing, transport, stability and translation.^9^, ^13^, ^50^ An increasing body of studies have demonstrated that various biological processes are tightly regulated by m^6^A modification, such as haematopoiesis, neurodevelopment and spermatogenesis.^18^, ^19^, ^51^, ^52^ Spermatogenesis is a complex and multi‐stage biological process involving self‐renewal and differentiation of spermatogonia, meiosis and spermiogenesis.^53^, ^54^ During spermatogenesis, the germ cells undergo dramatic changes in transcriptomic, epigenomic, proteomic and metabolic levels, with the biogenesis and removal of various biomolecules in distinct stages.^55^ Single‐cell RNA‐seq has shown that spermatogenic cells of different stages have their own unique transcriptomic characteristics, which are sophisticatedly regulated.^29^ YTHDF2, an m^6^A binding protein, is reported to be critical for mediating mRNA decay.^14^, ^45^ By deleting exon 2 of *Ythdf2*, an initial study found these conventional knockout males on a mixed genetic background possess normal reproductive capability,^26^ while in a later study, the authors used CRISRP/Cas9 to delete exon 4 of *Ythdf2*, and found that the surviving KO males on a mixed genetic background are subfertile.^25^ These findings indicate that different targeting strategies and genetic background might lead to distinct outcomes. In this study, we generated the germ cell–specific knockout mice on a C57BL/6 genetic background to dissect the exact role of YTHDF2 during spermatogenesis, and uncovered that the global transcriptional dysregulation may be the major cause that contributes to sperm deformity and infertility upon *Ythdf2* deletion. Mechanistically, we demonstrated that the proper and timely mRNA clearance mediated by YTHDF2 is essential for spermatogenesis.

As m^6^A ‘reader’ proteins, YTH family members have been shown to be crucial for mRNA processing, stability and translation. Interestingly, their expressions are quite dynamic in germ cells. During spermatogenesis, *Ythdc1* deletion results in progressive loss of spermatogonia.^20^ *Ythdc2* KO mice are infertile and exhibit meiotic arrest due to inefficient translation and degradation of YTHDC2‐targeted mRNA.^21^, ^22^ In our study, we showed that *Ythdf2* deletion has no effect on the self‐renew and differentiation of spermatogonia, but it is indispensable in later developmental stages of spermatogenesis. Our results showed that YTHDF2 is mainly expressed in spermatogonia and spermatocytes, and its expression is decreased rapidly in the later stages. We collected different stages of germ cells to perform RNA‐seq analysis, which showed that the dysregulated genes are significantly increased at spermatid. Given YTHDF2 expression is very low in spermatid, most likely, the transcriptomic disorder might be a secondary effect. Similarly, Tang et al. reported that ALKBH5 depletion results in abnormal spermatogenesis by affecting transcript changes at pachytene stage and round spermatid stage.^19^ Mice with inactivated both *Mettl3* and *Mettl14* in advanced germ cells with *Stra8*‐*Cre* show normal meiosis, decreased sperm count and destroyed sperm motility,^18^ which is very similar to the phenotype of our *Ythdf2*‐vKO and *Ythdf2*‐sKO mice, indicating that YTHDF2 is in charge of regulating the m6A‐decorated transcripts in late spermatogenesis.

An analysis of the m^6^A methylome of spermatogenic cells at different developmental stages uncovered that there are a lot of identical transcripts marked by m^6^A at several different stages.^18^ Our analysis of YTHDF2‐RIP‐seq at different developmental stages indicated that some of the above‐mentioned transcripts are also YTHDF2 targets at different stages, such as *Brwd1* and *Jarid2*. They are all expressed at both spermatogonia and pachytene spermatocyte, meanwhile, they are also targeted by YTHDF2 at both stages. It raises an open question concerning how YTHDF2 is switched on at the right time and place. An attractive hypothesis is that YTHDF2 may accelerate mRNA clearance only when it becomes redundant, which deserves further study.

## CONFLICT OF INTEREST

The authors declare that they have no conflict of interest.

## AUTHOR CONTRIBUTIONS

BS, HC and XJG conceived the experiments and designed the experiments. MQ performed all experiments with the help of BS, YG, YZ, XYG, XC and FW. HS and JJ performed all computational analyses. HM and XJG critically revised the manuscript. MQ, BS and HC wrote the manuscript. All authors contributed to the study and approved the submitted version.

## Supporting information

Supplementary Material

Table S2

Table S3

## Data Availability

The high‐throughput sequencing data have been deposited to the NCBI Sequence Read Archive (SRA) database (accession ID, PRJNA761327).
