# Supplementary material for: m6A reader protein YTHDF2 regulates spermatogenesis by timely clearance of phase‐specific transcripts
Source: Cell Prolif. 2021 Nov 30;55(1):e13164. doi: 10.1111/cpr.13164 (PMC8780898; doi:10.1111/cpr.13164)
Supplement: Supplementary file 1 — Supplementary Material [file CPR-55-e13164-s001.docx]

Supplementary information

**The m^6^A reader protein YTHDF2 regulates spermatogenesis by timely clearance of phase-specific transcripts**

**Contents**

**Figure S1.** *Ythdf2* expression in germ cells and its domain architecture.

**Figure S2.** Fertilization ability analysis of *Ythdf2*-vKO sperm.

**Figure S3.** YTHDF2 is dispensable for meiosis.

**Figure S4.** YTHDF2 is dispensable for spermatogonia.

**Figure S5.** Phenotype analysis of *Ythdf2*-sKO mice.

**Figure S6.** Gene expression analysis of germ cells at different stages.

**Figure S7.** Data quality analysis of YTHDF2 RIP-seq.

**Figure S8.** Analysis of delayed RNAs during spermatogenesis.

**Figure S9.** Overlaps of DEGs in round spermatid, m6A-modified and YTHDF2-targeted genes.

**Supplementary Table 1.** Information of primers.

**Supplementary Table 2.** List of DEGs in germ cells at different stages (excel file).

**Supplementary Table 3.** List of YTHDF2-targeted genes (excel file).


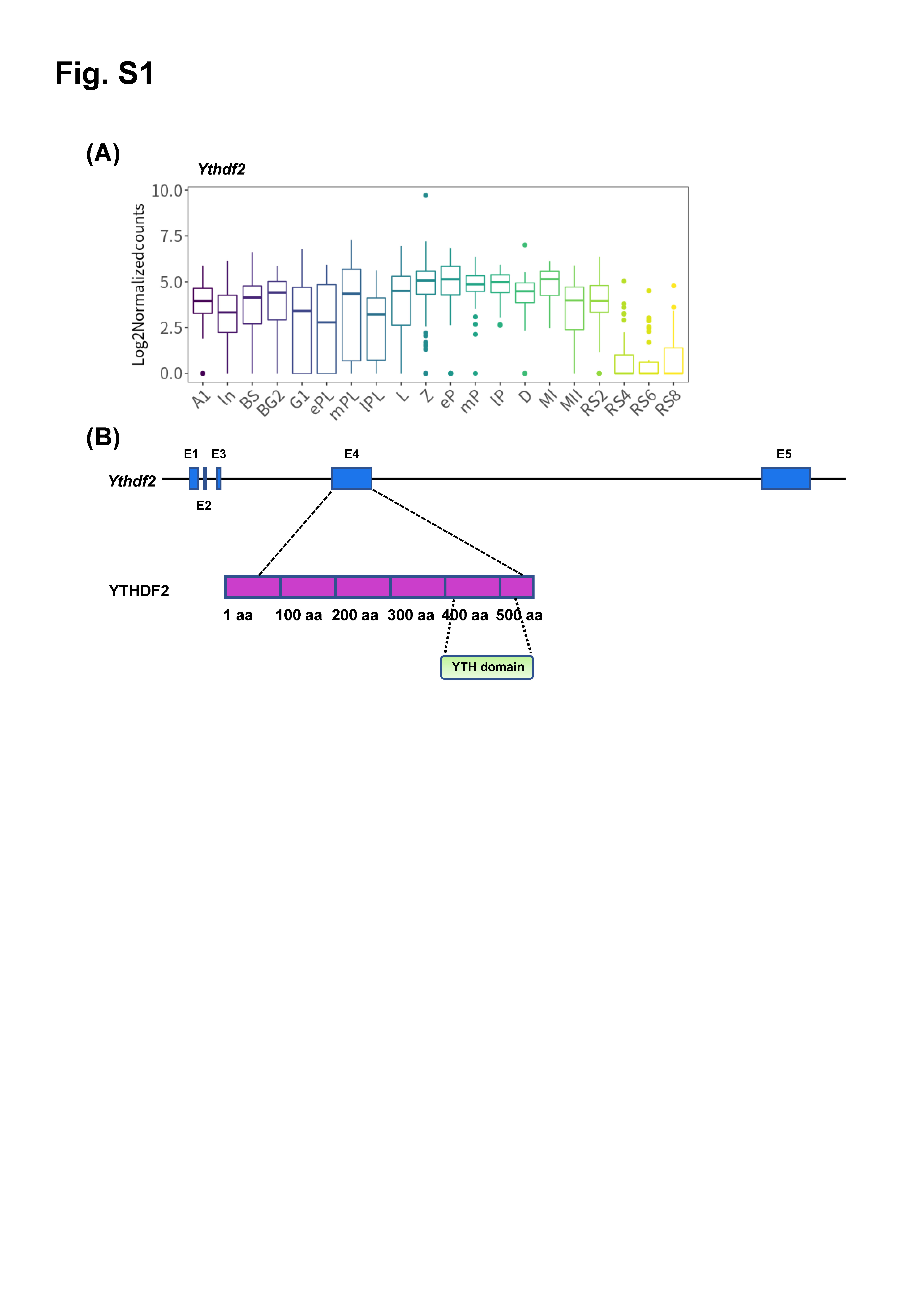


**Figure S1. *Ythdf2* expression in germ cells and its domain architecture.**

(A) Relative expression of *Ythdf2* during mouse spermatogenesis. It was acquired from a published single-cell RNA-seq data^1^

(B) A schematic representation of domain architecture of YTHDF2.


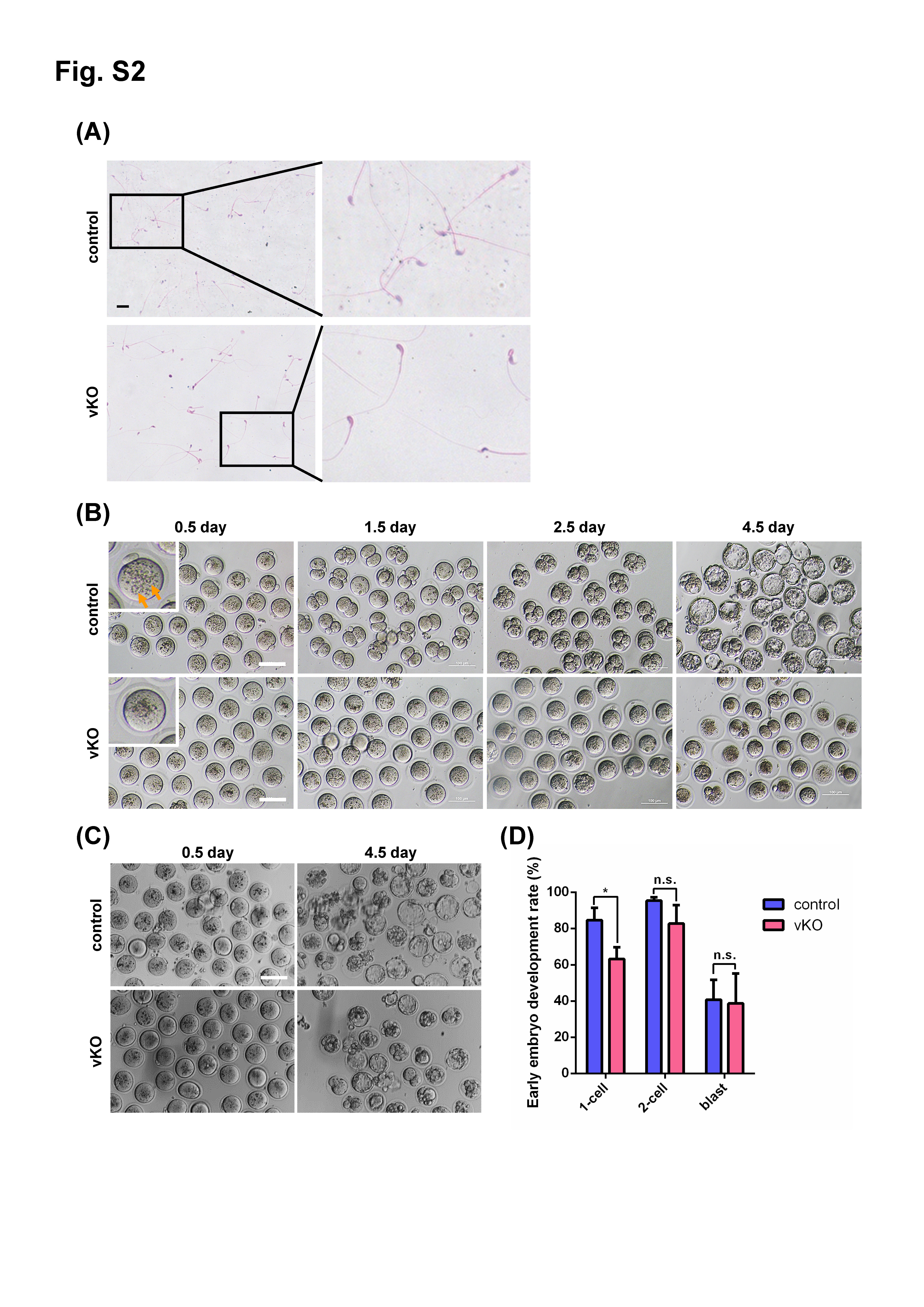


**Figure S2. Fertilization ability analysis of *Ythdf2*-vKO sperm.**

(A) Hematoxylin and eosin-stained sperm collected from control and *Ythdf2*-vKO cauda epididymides. Scale bar, 20 μm.

(B) Representative images of embryos after IVF using control and *Ythdf2*-vKO sperm. Scale bar, 100 μm. The orange arrows indicate the male and female pronucleus.

(C) Representative images of embryos after ICSI using control and *Ythdf2*-vKO sperm. Scale bar, 100 μm.

(D) Early embryo development rate analysis of ICSI. Data are presented as means ± SD (*n* = 3 for each group). Significance was calculated with unpaired two-tailed Student’s t-test (n.s., not significant, * *P* < 0.05).


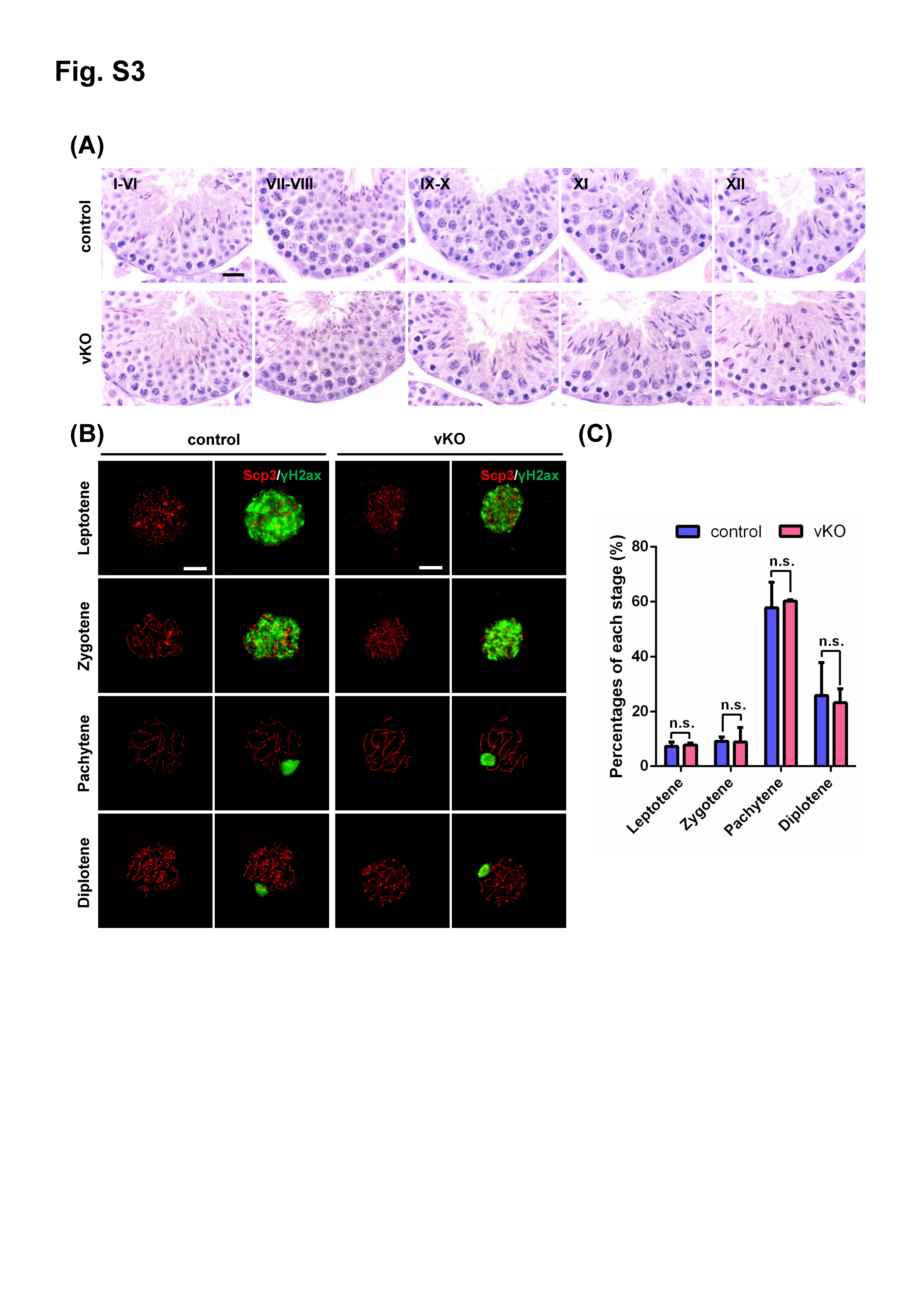


**Figure S3. YTHDF2 is dispensable for meiosis.**

(A) The cycle of the seminiferous epithelium in adult control and age-matched *Ythdf2*-vKO testes. Scale bar, 20 μm.

(B) Chromosome spreads of control and *Ythdf2*-vKO spermatocytes. Scale bars, 10 μm.

(C) Quantification of spermatocytes at different stages in adult control and age-matched *Ythdf2*-vKO testes. Data are presented as means ± SD (*n* = 3 for each group). Significance was calculated with unpaired two-tailed Student’s *t*-test (n.s., not significant).


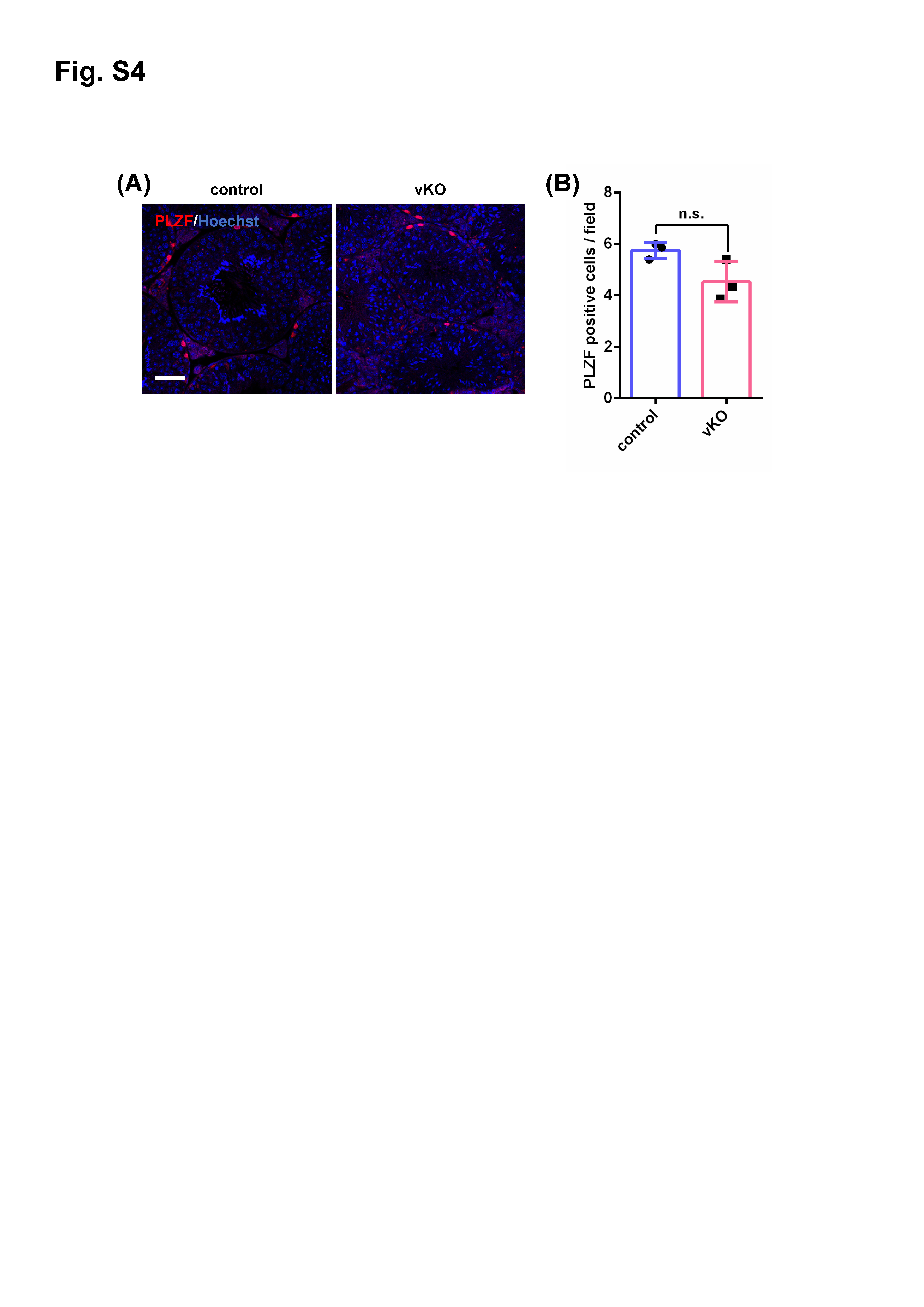


**Figure S4. YTHDF2 is dispensable for spermatogonia.**

(A) Immunofluorescence staining of PLZF (spermatogonia marker) in control and age-matched *Ythdf2*-vKO testes. Scale bar, 50 μm.

(B) Quantification of PLZF positive spermatogonia in control and age-matched *Ythdf2*-vKO testes. Data are presented as means ± SD (*n* = 3 for each group). Significance was calculated with unpaired two-tailed Student’s *t*-test (n.s., not significant).


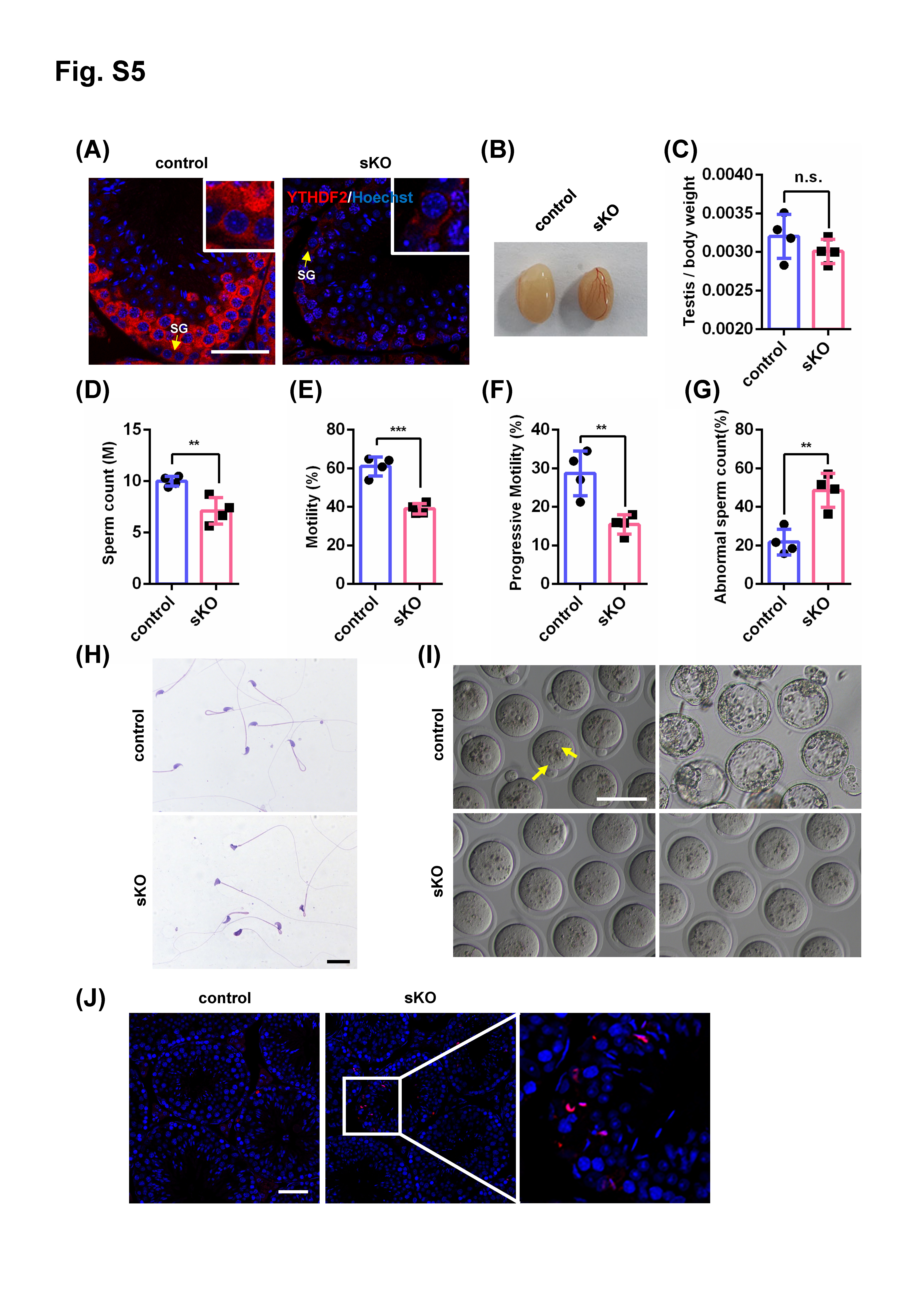


**Figure S5. Phenotype analysis of *Ythdf2*-sKO mice.**

(A) Immunofluorescent staining of YTHDF2 in adult control and *Ythdf2*-sKO testes. Scale bar, 50 μm. Yellow arrows indicate spermatogonia (SG).

(B) Gross morphology of representative testes from adult control and age-matched *Ythdf2*-sKO.

(C) The testis/body weight ratio in adult control and *Ythdf2*-vKO mice.

(D) The sperm count in cauda epididymides of adult control and age-matched *Ythdf2*-sKO mice. M, million. Data are presented as means ± SD (*n* = 4 for each group). Significance was calculated with unpaired two-tailed Student’s *t*-test (** *P* < 0.01).

(E) and (F) CASA assay of motility and progressive motility of sperm from adult control and age-matched *Ythdf2*-sKO mice.

(G) The percentage of abnormal sperm in adult control and *Ythdf2*-vKO mice.

(H) Hematoxylin and eosin-stained sperm collected from control and *Ythdf2*-sKO cauda epididymides. Scale bar, 20 μm.

(I) Representative images of embryos after IVF using control and *Ythdf2*-sKO sperm. Scale bar, 100 μm. The yellow arrows indicate the male and female pronucleus.

(J) TUNEL assay of histological cross-sections adult control and age-matched *Ythdf2*-vKO testes. Scale bar, 50 μm.

Data of C-G are presented as means ± SD (*n* = 4 for each group). Significance was calculated with unpaired two-tailed Student’s *t*-test (n.s., not significant, ** *P* < 0.01, *** *P* < 0.001).


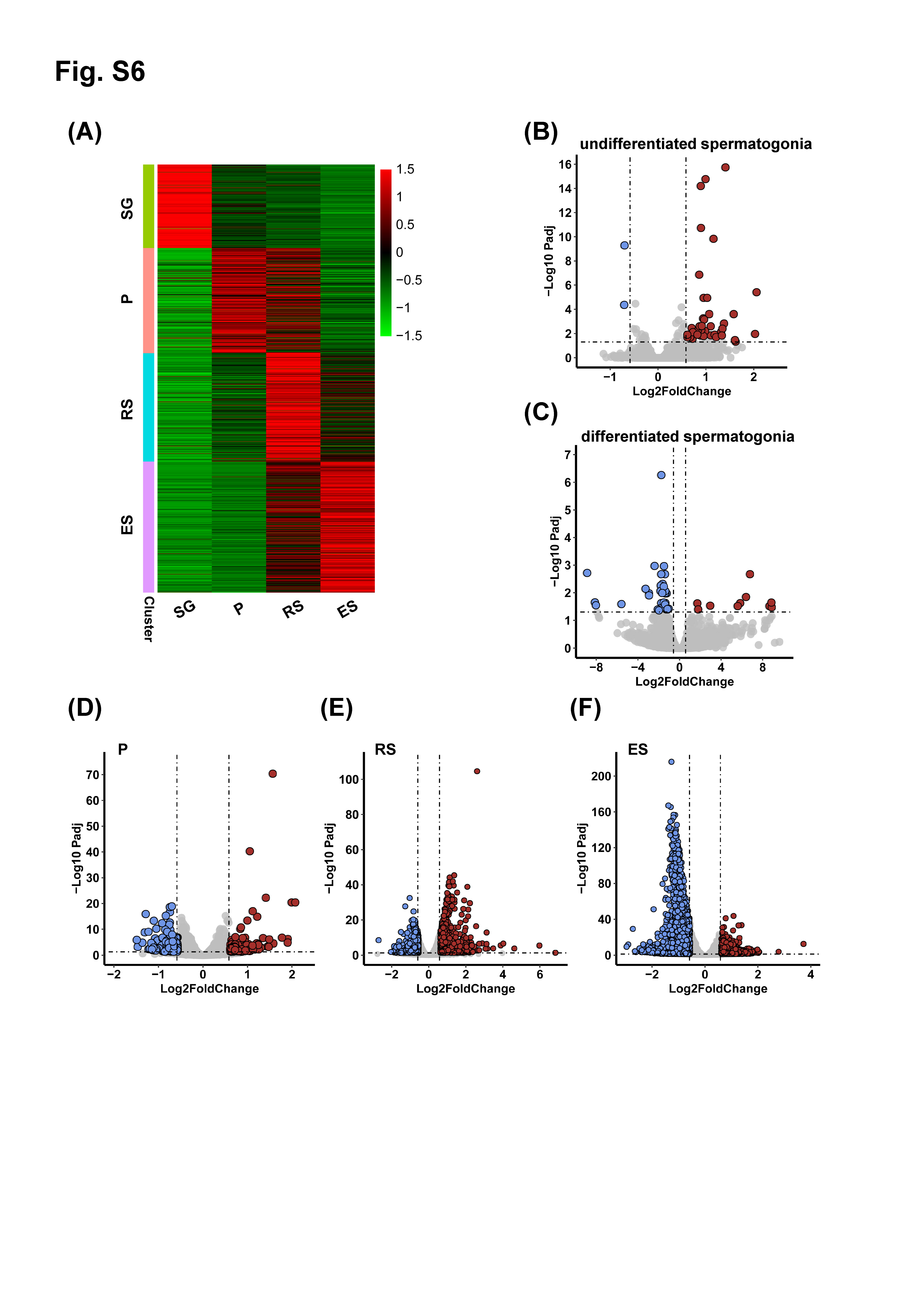


**Figure S6. Gene expression analysis of germ cells at different stages.**

(A) Heatmap of expression of marker genes at various stages of germ cells. The marker genes of different stages are identified by a previous Single-cell RNA-seq analysis^2^.

(B) to (F) Differential expressed genes (DEGs) analysis of *Ythdf2*-vKO germ cells at various stages compared with the control. Down-regulated genes are indicated by blue dots, Up-regulated genes are indicated by red dots. P, pachytene spermatocyte, RS, round spermatid, ES, elongated spermatid.


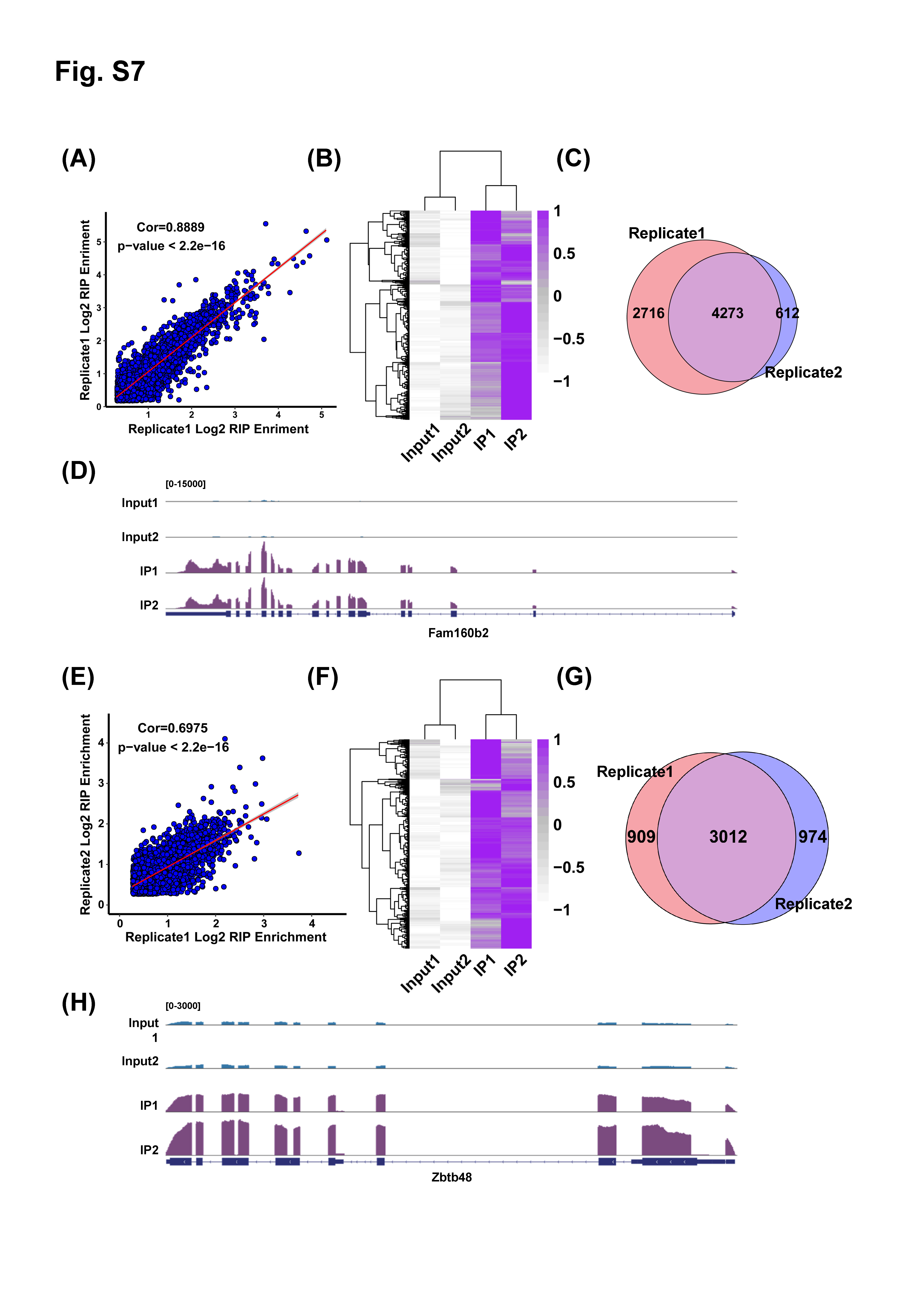


**Figure S7. Data quality analysis of YTHDF2 RIP-seq.**

(A) Correlation of two replicates of YTHDF2-targeted transcripts from adult whole testes.

(B) Heatmap of YTHDF2-targeted genes of adult whole testes.

(C) Venn diagrams show the overlaps of two replicates of YTHDF2-targeted transcripts from whole testes.

(D) YTHDF2-RIP read coverage from adult whole testes along *Fam160b2* genomic locus is shown in pink. Input coverage is shown in blue.

(E) Correlation of two replicates of YTHDF2-targeted transcripts from pachytene spermatocytes.

(F) Heatmap of YTHDF2-targeted genes from pachytene spermatocytes.

(G) Venn diagrams show the overlaps of two replicates of YTHDF2-targeted transcripts from pachytene spermatocytes.

(H) YTHDF2-RIP read coverage from pachytene spermatocytes along *Zbtb48* genomic locus is shown in pink. Input coverage is shown in blue.


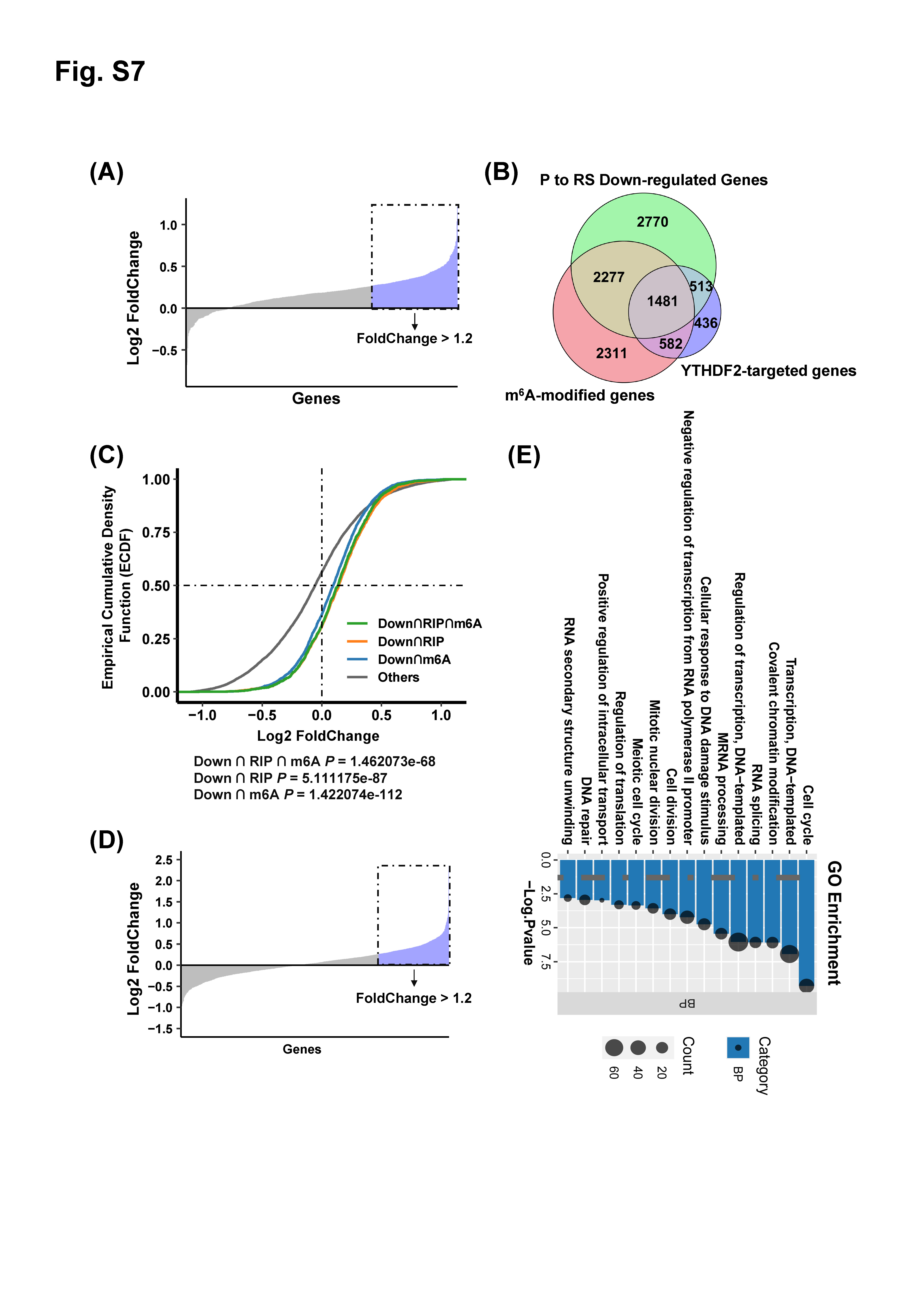


**Figure S8. Analysis of delayed RNAs during spermatogenesis.**

(A) The expression change of 736 shared genes from Figure 4A in *Ythdf2*-vKO pachytene spermatocytes relative to control.

(B) Venn diagram showing that overlaps among genes down-regulated from control pachytene spermatocyte to round spermatid, m6A-modified genes and YTHDF2-targeted genes. The m6A-seq data was acquired from a published data^3^. YTHDF2-targeted genes were identified in wild-type pachytene spermatocytes.

(C) Cumulative distributions of relative stability change between control and *Ythdf2*-vKO round spermatids.

(D) The RNA expression change of 1481 genes from Figure S7B in *Ythdf2*-vKO round spermatids relative to control.

(E) GO analysis of genes with delayed degradation from pachytene spermatocyte to round spermatid.


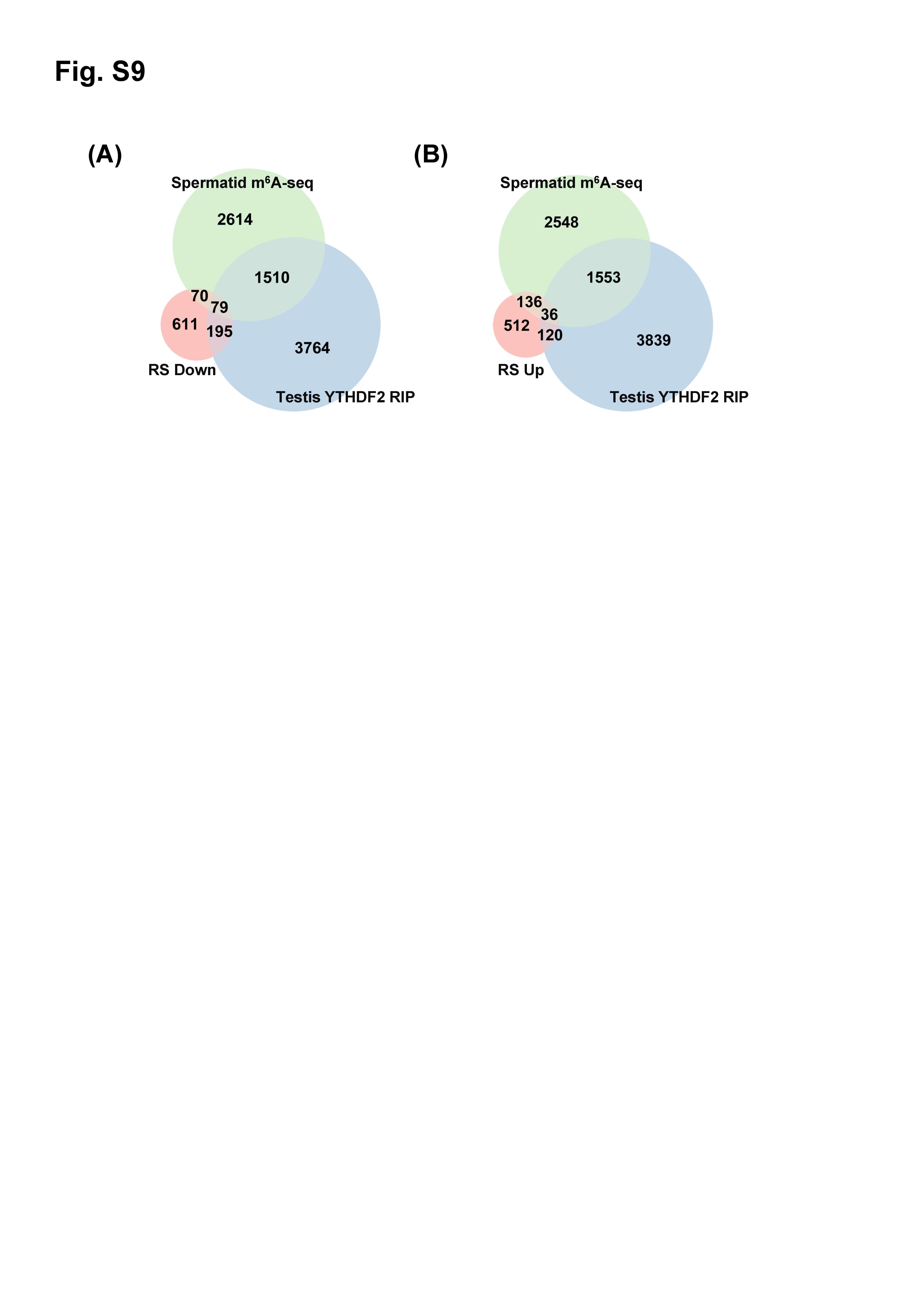


**Figure S9.** **Overlaps of DEGs in round spermatid, m6A-modified and YTHDF2-targeted genes.**

(A) Venn diagram showing that overlaps of down-regulated genes in round spermatid, m6A-modified genes and YTHDF2-targeted genes. The m6A-seq data was acquired from a published data3. YTHDF2-targeted genes were identified in adult whole testes.

(B) Venn diagram showing that overlaps of up-regulated genes in round spermatid m6A-modified genes and YTHDF2-targeted genes.

**Supplementary Table 1.** Information of primers.

| **Gene** | **Forward sequence (5' - 3')** | **Reverse sequence (5' - 3')** |
| --- | --- | --- |
| *Brwd1* | AACGGCTGCACATCCCACACTG | CTGCCCTGAGTTTCTGCCCAGA |
| *Jarid2* | GCAAGTGGACAAGCCGCAGTCA | CGCTCGGGCACTTCCTTCTTCA |
| *Egr1* | CAGCAGCTCCTTCAGCACCTCA | TTGCGGCCATCTCTTCCCTCCT |
| *Tsc22d3* | ATGCCCATCTGGGTCCACTCCA | ACGACAGGCTCACTGGCTTGGT |
| *Cluc* | GCTTCAACATCACCGTCATTG | CACAGAGGCCAGAGATCATTC |
| *Gapdh* | AGGTCGGTGTGAACGGATTTG | TGTAGACCATGTAGTTGAGGTCA |

**Reference**

1. Chen Y, Zheng Y, Gao Y, et al. Single-cell RNA-seq uncovers dynamic processes and critical regulators in mouse spermatogenesis. *Cell Res*. 2018; **28**(9): 879-896.

2. Green CD, Ma Q, Manske GL, et al. A Comprehensive Roadmap of Murine Spermatogenesis Defined by Single-Cell RNA-Seq. *Dev Cell*. 2018; **46**(5): 651-667 e610.

3. Lin Z, Hsu PJ, Xing X, et al. Mettl3-/Mettl14-mediated mRNA N(6)-methyladenosine modulates murine spermatogenesis. *Cell Res*. 2017; **27**(10): 1216-1230.
